# MDL-CA: a multimodal deep learning approach with a cross attention mechanism for accurate brain cancer diagnosis

**DOI:** 10.3389/fpubh.2025.1687335

**Published:** 2026-01-05

**Authors:** Sumaira Sarwar, Saqib Majeed, Asif Nawaz, Ruqia Bibi, Seung Won Lee

**Affiliations:** 1 University Institute of Information Technology, PMAS-Arid Agriculture University Rawalpindi, Rawalpindi, Pakistan; 2 Department of Precision Medicine, Sungkyunkwan University School of Medicine, Suwon, Republic of Korea; 3 Department of Artificial Intelligence, Sungkyunkwan University, Suwon, Republic of Korea; 4 Department of MetaBioHealth, Sungkyunkwan University, Suwon, Republic of Korea; 5 Personalized Cancer Immunotherapy Research Center, Sungkyunkwan University School of Medicine, Suwon, Republic of Korea; 6 Department of Family Medicine, Kangbuk Samsung Hospital, Sungkyunkwan University School of Medicine, Seoul, Republic of Korea

**Keywords:** 3D DenseNet, brain cancer, deep learning, Entmax, GAT, multimodality

## Abstract

**Introduction:**

Brain cancer diagnosis poses a significant clinical challenge due to the complex interplay between molecular mechanisms and anatomical abnormalities. Traditional diagnostic techniques, including invasive biopsies, isolated genomic assays, and standalone Magnetic Resonance Imaging (MRI), often exhibit limitations such as procedural risks, inadequate sensitivity, and incomplete assessment of tumor heterogeneity. These shortcomings contribute to delayed diagnosis, inaccurate tumor grading, and suboptimal treatment planning. Furthermore, single-modality data, whether MRI or genomic profiles, frequently yield limited diagnostic accuracy and biological interpretability.

**Methods:**

To address these limitations, this study proposes MDL-CA, a Multimodal Deep Learning framework with a Cross-Attention mechanism, designed to integrate genomic and MRI modalities for enhanced brain cancer diagnosis. The framework fuses genomic graph embeddings, extracted using a Graph Attention Network (GAT), with MRI feature maps derived from a 3D DenseNet. The cross-modal attention fusion mechanism enables the model to capture intricate biological and spatial interactions, producing a biologically informed feature representation. Additionally, the Entmax sigmoid function is employed in the classification stage to promote sparsity and improve interpretability. Data were sourced from The Cancer Imaging Archive (TCIA) and The Cancer Genome Atlas (TCGA) following comprehensive preprocessing.

**Results:**

Extensive experiments conducted across four benchmark datasets demonstrated that MDL-CA achieved superior diagnostic performance, with accuracies of 96.22%, 97.14%, 98.46%, and 98.21%, and F1-scores ranging from 95.95% to 98.40%. These results confirm the framework’s robustness, scalability, and consistent generalization across diverse datasets.

**Discussion:**

The integration of genomic and MRI data through the proposed cross-attention mechanism enables deeper biological understanding and improved diagnostic precision compared to single-modality and conventional fusion approaches. By effectively modeling interactions between molecular and anatomical features, MDL-CA advances the development of biologically informed, multimodal diagnostic systems for brain cancer. The results highlight the framework’s potential to support early diagnosis and personalized treatment planning in clinical practice.

## 1 Introduction

Brain cancer refers to tumors that arise from the abnormal and continuous growth of cells within the brain (1). Tumors can be either primary, originating in the brain, or secondary, indicating metastasis from other organs. Brain cancer encompasses a wide range of tumor classes, with glioblastomas and gliomas being among the most prevalent and locally aggressive types (2). These tumors exert pressure on surrounding healthy tissue, disrupting normal brain function and leading to neurological symptoms such as headaches, seizures, cognitive decline, and motor impairments. The diagnosis and treatment of brain cancer are complicated by its heterogeneous cellular structure. Tumor location and size significantly influence prognosis; therefore, early detection and precise diagnosis are essential for improving patient outcomes (3). Globally, approximately 300,000 people are affected by brain cancer each year, with over 80,000 new cases reported annually in the United States (4). Despite advances in medical technology, the survival rates for certain brain cancers, particularly glioblastoma, remain critically low. This underscores the urgent need for more effective diagnostic techniques and improved treatment options.

The rising incidence of brain cancer has fueled the demand for more accurate diagnostic tools capable of achieving reliable tumor classification, early detection, and robust outcome prediction. Recent advances in machine learning (ML) have opened new possibilities for analyzing complex multimodal data and enhancing diagnostic accuracy (5). Genomic data plays a crucial role in brain cancer diagnosis by offering detailed insights into the molecular alterations that drive tumor development. Tumor types such as gliomas and glioblastomas can be distinguished based on copy number alterations, gene expression variations, and specific genetic mutations, which serve as biomarkers. Examining a tumor’s genetic profile provides valuable information about its behavior, aggressiveness, and potential therapeutic response. Identifying targetable mutations also enables personalized treatment, thereby improving patient outcomes (6). Integrating genomic data with diagnostic imaging supports more accurate and individualized treatment planning, going beyond traditional imaging-based approaches. MRI remains indispensable for visualizing the anatomical characteristics of brain tumors. MRI scans depict brain structure in detail, allowing clinicians to assess tumor location, size, shape, and interaction with surrounding tissues (7). Advanced MRI techniques, such as contrast-enhanced imaging, reveal vascular characteristics associated with tumor aggressiveness. When combined with genomic information, MRI offers a holistic diagnostic perspective, linking structural and molecular insights to support timely and precise decision-making (8).

Recent progress in multimodal data analysis has demonstrated that integrating genomic profiles with medical imaging enhances diagnostic performance. Deep learning (DL) models, particularly Convolutional Neural Networks (CNNs), have shown strong capability in extracting intricate patterns from MRI and other imaging modalities, enabling automated tumor characterization based on features such as shape, texture, and spatial distribution. Integrating these image-based features with genomic data (including mutations and gene expression patterns) provides a more comprehensive understanding of tumor biology (9). For instance, while MRI reveals tumor morphology, genomics identifies key molecular alterations. This synergy improves diagnostic precision, risk assessment, and treatment planning.

Consequently, multimodal deep learning approaches have emerged as powerful tools for personalized, data-driven decision-making in brain cancer care. In this domain, machine learning techniques such as Support Vector Machines (SVMs), Random Forests (RFs), and Gradient Boosting have been widely employed (10). These algorithms effectively handle structured genomic data to identify patterns that predict tumor subtypes and therapeutic responses. Classical ML methods, combined with feature extraction techniques (e.g., texture analysis, tumor segmentation), have also been used to analyze MRI data for classification and risk evaluation (11).

Deep learning models, especially CNNs, are particularly effective for processing complex imaging data such as MRI scans. CNNs can automatically learn hierarchical features from raw images, minimizing the need for manual feature engineering. When genomic data is incorporated, multi-input neural networks and deep multimodal architectures can jointly analyze both data types, revealing intricate associations between genetic alterations and tumor imaging features. These models aim to achieve accurate and reliable predictions, facilitating early detection and personalized treatment strategies (12).

Despite these advances, existing machine learning and deep learning approaches face substantial challenges when dealing with multimodal datasets combining genomic and MRI data. Traditional models often lack the capability to efficiently integrate heterogeneous data types, leading to suboptimal performance (13). The high dimensionality of genomic data increases the risk of overfitting, while MRI analysis requires complex and time-consuming feature extraction that may overlook critical patterns (14). Furthermore, most current models rely heavily on supervised learning, which depends on large, well-labeled datasets—often unavailable for rare brain cancer subtypes (15).

Therefore, there is a pressing need for a hybrid deep learning model capable of seamlessly integrating genomic and MRI data without extensive preprocessing or manual feature engineering. Such a model would harness the complementary strengths of both modalities to achieve more accurate tumor characterization and improved predictive outcomes. By leveraging modified multimodal neural network architectures, this approach can overcome existing limitations and provide a robust diagnostic framework for brain cancer (16). In summary, brain cancer remains a major healthcare challenge due to its biological complexity and the limitations of current diagnostic methods. While genomic data and MRI each provide valuable insights, their independent use within conventional machine learning frameworks has not achieved the level of diagnostic accuracy required for precise treatment planning. Deep learning-based multimodal models offer a promising solution by enabling the effective integration of imaging and genomic data, thereby enhancing early diagnosis, risk stratification, and personalized treatment for brain cancer patients.

To address the limitations of single-modality diagnostic models and the insufficient integration of molecular and anatomical information in existing multimodal approaches, this study proposes MDL-CA, a novel multimodal deep learning framework. The key innovation of MDL-CA lies in its cross-attention fusion mechanism, which injects genomic graph embeddings directly into intermediate MRI feature maps to capture biologically meaningful interactions that conventional fusion methods overlook. The primary objective of this research is to develop a biologically informed diagnostic model that enhances tumor characterization and improves classification accuracy. Specifically, this study aims to (i) integrate MRI and genomic modalities through a cross-modal attention mechanism, (ii) leverage graph-based genomic embeddings for interpretable molecular representation, and (iii) demonstrate the effectiveness and robustness of the proposed approach across multiple brain cancer datasets.

### 1.1 Research contribution

The key contributions of this work are as follows:

- This study highlights the critical limitations of existing brain cancer diagnostic systems that rely solely on either imaging or genomic data. It emphasizes the necessity of a multimodal approach capable of capturing both anatomical and molecular characteristics for improved diagnostic accuracy.
- The proposed MDL-CA framework integrates MRI and genomic data using a cross-modal attention fusion mechanism, enabling the direct embedding of genomic graph representations into MRI feature maps to capture complex spatial–biological relationships.
- This work leverages 3D DenseNet and GAT architectures for modality-specific feature extraction. Furthermore, it employs the Entmax sigmoid function for sparse and interpretable classification, resulting in biologically informed representations and enhanced model transparency.
- Extensive experiments conducted on four benchmark datasets demonstrate that MDL-CA achieves state-of-the-art performance, with accuracies of 96.22, 97.14, 98.46, and 98.21%, and F1-scores ranging from 95.95 to 98.40%, confirming the robustness and generalizability of the proposed approach.

The remainder of this paper is organized as follows. Section 2 presents the literature review and identifies gaps in existing multimodal models. Section 3 describes the methodology of the proposed approach, while Section 4 reports the experimental results and performance evaluation. Section 5 discusses the recommendations and practical implications of MDL-CA, and Section 6 concludes the study and outlines directions for future research.

## 2 Literature review

This section reviews recent research on the use of machine learning (ML) and deep learning (DL) techniques in brain cancer diagnosis, with a particular focus on the integration of genomic and MRI data. It critically examines existing studies, highlighting their strengths, limitations, and the challenges associated with analyzing multimodal datasets. Table 1 summarizes the key works in this domain, identifying their methodological advantages and drawbacks. Additionally, this section discusses emerging models that aim to overcome current barriers and improve diagnostic accuracy. To establish the foundation for the proposed approach, we also review relevant studies addressing the identified research gaps.

**Table 1:** Summarized literature review on cancer diagnosis using multimodality.

References	Core method	Obtain accuracy	Major limitations
(28)	Multimodal Fusion Deep Neural Network (MFDNN) combining imaging, genomics, and clinical data	92.5% accuracy, 87.4% precision, 86.4% recall, 86.2% F1-score	Requires substantial multimodal data for effective performance, and ethical concerns on AI deployment.
(29)	Multimodal deep learning framework for cervical cancer using PET/CT and other images.	6.06% improvement over PET, 8.9% improvement over other multimodal fusion methods.	Focuses only on image fusion and detection. Lacks broader applicability.
(30)	Deep learning model integrates WL and IEE images for CRC invasion depth estimation.	91.61% (internal test) and 93.78% (without advanced CRC), 100% in video test.	Limited to CRC. Not applicable on other cancer types.
(31)	Multimodal data (CT, ultrasound) combined for predicting metastasis in ccRCC.	AUC of 0.924 (training), 0.877 (internal), 0.849 (external) for predicting LNM.	Relies on data from specific regions May not generalize to all populations.
(32)	Radiomics model using multimodal MRI for predicting post-treatment response of LCBM.	AUC of 0.930 (primary cohort), 0.852 (validation cohort), C index 0.930.	Does not incorporate other modalities like genomics. Limited to imaging features.

Ye (17) discussed recent advances in diagnostic and treatment technologies that enable precision radiotherapy for glioblastoma. The authors emphasized the integration of biologically informed multimodality imaging to address the spatial and temporal heterogeneity underlying treatment resistance. They highlighted that several candidate imaging biomarkers have emerged beyond traditional diagnostic utility, with early-phase clinical trials demonstrating both safety and potential therapeutic benefits. Furthermore, the study proposed a rationale for implementing advanced MRI and positron emission tomography (PET) biomarkers to guide personalized radiotherapy. The authors suggested that response-adaptive radiotherapy, guided by biologically informed imaging, could improve outcomes in glioblastoma by supporting individualized treatment strategies in combination with novel systemic therapies.

Hölscher and Bülow (18) explored the rapidly growing field of computational pathology, which has shown strong potential for developing objective prognostic models based on histopathological images. The authors noted that most existing approaches rely exclusively on either histology or genomic data, without integrating these modalities to form joint image-omic prognostic frameworks. To address this limitation, they proposed a multimodal deep learning model that jointly analyzes whole-slide pathology images and molecular profiles across 14 cancer types. Their weakly supervised algorithm effectively fused these heterogeneous data sources to predict patient outcomes and identify prognostic features correlated with survival. Additionally, the authors presented a publicly accessible database to support further research and biomarker discovery, emphasizing the potential of explainable morphological and molecular descriptors to enhance understanding of cancer prognosis.

Ouyang et al. (19) investigated deep learning models for cancer prognosis that leverage multimodal data, incorporating molecular and histo-morphological information. The authors observed that prior approaches often fail to comprehensively capture biological and histological relationships, and rarely utilize pretraining strategies that could enhance performance. To address these shortcomings, they developed an interpretable multimodal framework integrating DNA methylation, gene expression, and histopathology data. The study compared cross-modal pretraining, contrastive learning, and transfer learning techniques against standard baselines, demonstrating that their models significantly outperformed state-of-the-art methods and clinical benchmarks, achieving substantial improvements in C-index scores. Moreover, model interpretability facilitated the identification of biologically meaningful prognostic factors, underscoring the importance of pretraining strategies and tumor microenvironment considerations in accurate prognosis prediction.

Mahootiha et al. (20) focused on Glioblastoma (GBM), the most common and lethal malignancy of the central nervous system, characterized by high heterogeneity and frequent recurrence. In their review, the authors emphasized the growing importance of molecular subtyping in precision medicine, which enables characterization of the cellular and genetic complexity of GBM and its resistance to therapy. They summarized recent research proposing tetra-fractional and tripartite methods for detecting molecular subtypes, each exhibiting distinct gene expression patterns and biological behaviors. Furthermore, the study described how GBM subtypes display regulatory plasticity, oncogene variability, microenvironmental changes, and differential therapeutic responses. The authors also discussed mesenchymal transition mechanisms driven by diverse regulatory factors, highlighting the importance of a deep understanding of subtype-specific molecular features to inform targeted treatment strategies.

**Figure 1:**
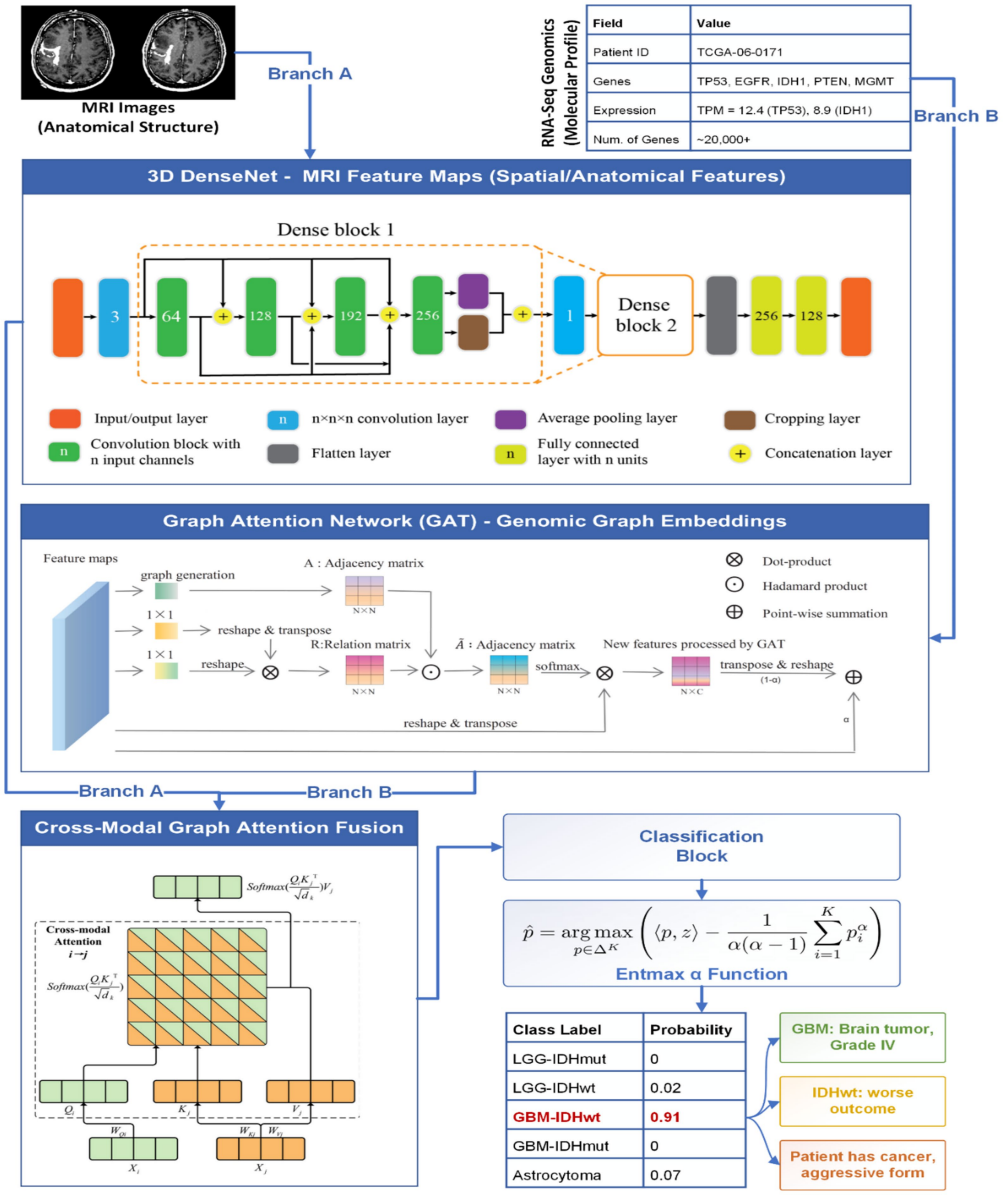
Proposed MDL-CA model for brain cancer prediction.

Yu et al. (21) proposed an innovative machine learning approach to identify genetic variants associated with brain structure and function using neuroimaging data. The authors noted that conventional genome-wide association studies (GWASs) in neuroimaging genetics typically analyze univariate quantitative features derived from brain images, limiting their scope. To advance this field, they implemented a deep learning framework capable of classifying MRI brain images based on single nucleotide polymorphism (SNP) genotypes. They hypothesized that if MRI images labeled by specific SNP genotypes could be reliably distinguished through ML, these variants would likely be associated with neuroanatomical or functional differences. Applying this methodology to the Alzheimer’s Disease Neuroimaging Initiative (ADNI) dataset, they identified novel variants with strong associations to brain phenotypes, demonstrating the potential of deep learning to uncover genetic determinants of brain imaging traits (Figure 1).

Although multimodal deep learning models show strong potential for cancer diagnosis, several challenges persist. Current research often focuses on individual cancer types and faces limited generalizability, frequently overlooking genomic and molecular data. Furthermore, many models rely heavily on large, high-quality datasets, which are rarely available in clinical settings. In the context of brain cancer, integrating multiple data sources, particularly genomics and imaging, is crucial to ensure accurate diagnosis. Therefore, there is a clear need for a novel multimodal approach that effectively addresses these limitations and enhances both diagnostic accuracy and treatment planning.

## 3 Materials and methods

**Figure 2:**
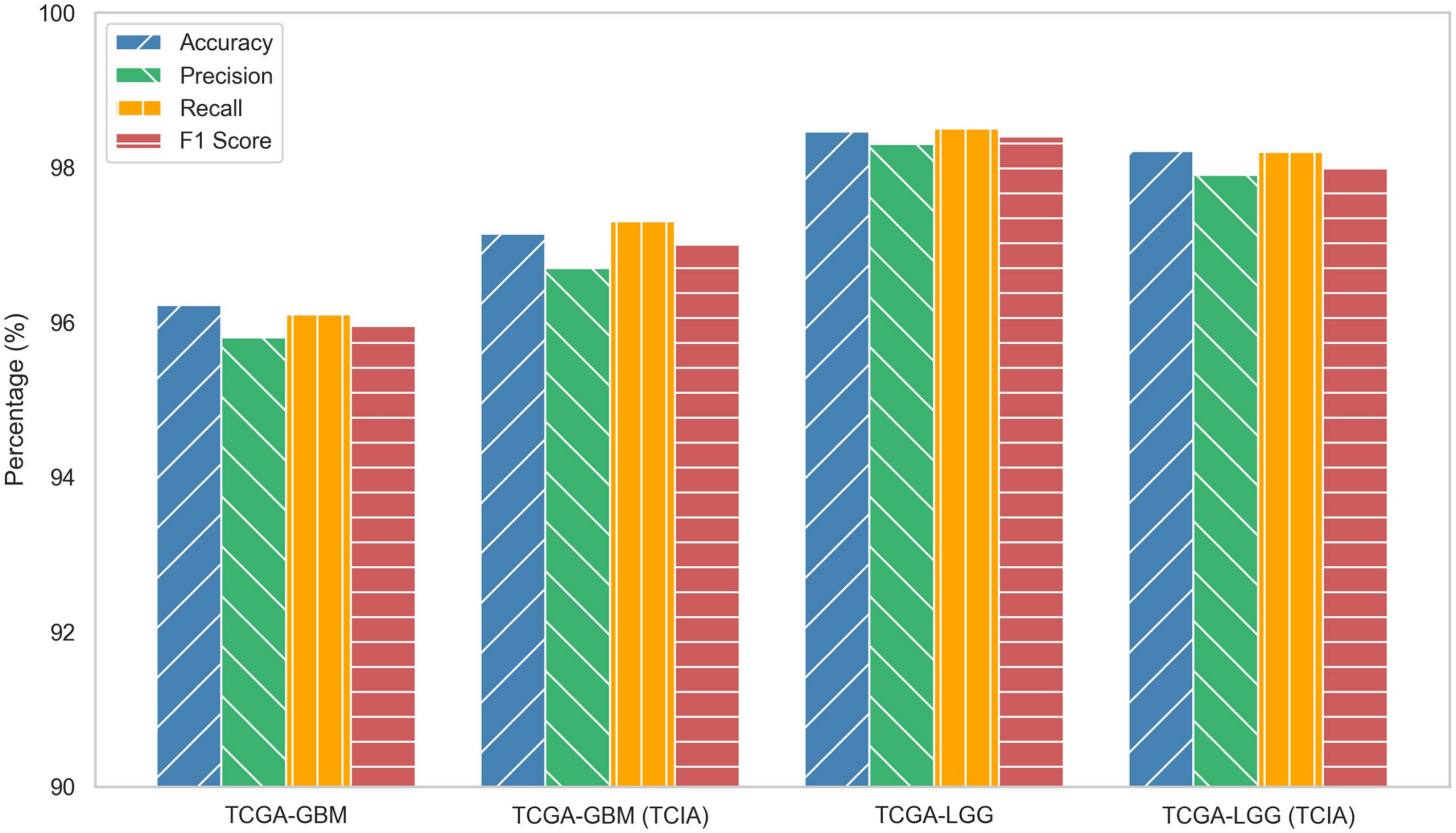
Performance evaluation for MDL-CA model.

The proposed methodology, as illustrated in Figure 2, comprises several key stages, including data collection, preprocessing, feature extraction and fusion, fusion optimization, and final classification. A detailed description of each algorithmic step is provided in the following subsections.

### 3.1 Data collection

The TCIA is a publicly accessible and well-organized repository that provides extensive cancer imaging data, including brain MRI scans from over 2,000 patients (as listed in Table 1). Collections such as TCGA-GBM and TCGA-LGG (referenced in Table 1) offer multimodal imaging data linked with corresponding genomic profiles, making TCIA a valuable resource for developing and validating artificial intelligence (AI) models in medical imaging and cancer analysis.

The TCGA serves as a pivotal resource that provides multi-omics data, including genomic, transcriptomic, and proteomic profiles for over 11,000 patients across more than 30 cancer types. It includes detailed brain tumor datasets encompassing approximately 1,200 patients, such as those with glioblastoma multiforme (GBM) and lower-grade glioma (LGG). Each case is accompanied by clinical metadata linking molecular profiles to treatment information and patient outcomes, thereby supporting integrative cancer research and the development of multimodal diagnostic frameworks.

Table 2 outlines the multimodal brain cancer datasets sourced from TCGA and TCIA, emphasizing their utility for integrative research. It includes genomic datasets such as TCGA-GBM and TCGA-LGG, comprising 200 and 290 patient records, respectively, along with matched MRI scans obtained from TCIA. These datasets enable combined molecular and imaging analysis, supporting the development of robust multimodal deep learning frameworks for brain cancer diagnosis and prognosis. To correctly pair MRI and genomic features, each MRI volume was associated with its corresponding genomic profile at the patient level using TCGA/TCIA patient IDs. Only samples with both modalities available were included in the multimodal experiments. When MRI scans contained multiple sequences, T1c was selected for consistency. Genomic features were normalized per patient and synchronized with MRI-level batches during training by repeating the genomic embedding for each MRI patch belonging to the same patient. Each dataset entry specifies the number of records and access links, providing a transparent and reproducible foundation for further experimentation.

**Table 2:** Datasets sources.

Dataset name	Description	Number of records	Access link
TCGA GBM	Glioblastoma Multiforme dataset containing genomic data for primary glioblastoma samples.	206 tumor and matched control pairs.	https://portal.gdc.cancer.gov/projects/TCGA-GBM
TCGA-LGG	Lower Grade Glioma dataset with genomic data for lower grade glioma samples.	293 tumor and matched control pairs.	https://portal.gdc.cancer.gov/projects/TCGA-LGG
TCGA-GBM (TCIA)	MRI imaging dataset corresponding to TCGA GBM genomic data, including various MRI sequences.	206 imaging studies.	https://www.cancerimagingarchive.net/collection/tcga-gbm/
TCGA-LGG (TCIA)	MRI imaging dataset corresponding to TCGA LGG genomic data, including various MRI sequences.	293 imaging studies.	https://www.cancerimagingarchive.net/collection/tcga-lgg/

For each dataset, we provide detailed information regarding the class distribution to ensure transparency. The TCGA–TCIA multimodal cohort included 214 patients with paired MRI–genomic data and three tumor classes: Glioblastoma Multiforme (GBM), Low-Grade Glioma (LGG) and Oligodendroglioma. The distribution was mildly imbalanced, with GBM comprising the largest portion. Additionally, the MRI-only datasets demonstrated similar imbalance patterns in high-grade versus low-grade tumor categories. To address this imbalance, we applied class-weighted loss functions and data augmentation (random rotations, contrast adjustments, and elastic deformations), ensuring that under-represented classes contributed adequately during training. Additionally, the class distribution analysis revealed mild imbalance across tumor categories, particularly in the multimodal cohort where GBM cases were more prevalent. This imbalance was addressed through a combination of class-weighted loss functions and targeted augmentation of minority classes. By reporting the distributions and mitigation strategies, we ensure that the experimental results are correctly contextualized and the applied methods are reproducible.

### 3.2 Data preprocessing

Algorithm 1 illustrates the sequential steps performed in the proposed model, emphasizing that data preprocessing is an essential phase in deep learning, particularly when handling multimodal medical inputs. This stage transforms raw data into standardized, noise-free formats, thereby enhancing model interpretability and learning efficiency. In brain cancer research, preprocessing is especially critical due to the heterogeneous nature of MRI and genomic data, each of which requires specialized treatment to ensure consistency, accuracy, and alignment across modalities.

For MRI data, preprocessing begins with skull-stripping, a procedure that removes non-brain tissues to isolate the brain region of interest (ROI). Let I(x,y,z) denote the original 3D MRI volume, and M(x,y,z) represent a binary brain mask obtained through segmentation algorithms. The skull-stripped brain image, $I_{\text{Brain}}(x,y,z),$ is then computed using Equation 1.


$$I_{\text{brain}}(x,y,z)=I(x,y,z)\times (x,y,z)$$ (1)

Next, intensity normalization is applied to standardize voxel intensity values, ensuring consistent contrast and brightness across MRI scans. This process typically employs z-score normalization, as expressed in Equation 2:


$$I_{\mathrm{norm}}=\frac{\left(I\text{brain}-\mu \right)}{\sigma }$$ (2)

The mean (*μ*) and standard deviation (*σ*) of the brain voxels are computed during normalization to ensure intensity standardization across samples. Subsequently, all MRI volumes are resized to a uniform shape of S×S×S(i.e.128×128×128) using an interpolation function $f_{\text{resize}}$ , as expressed in Equation 3. This step ensures consistent input dimensions for the 3D DenseNet architecture.


$$I_{\text{final}}=f_{\text{resize}}(I_{\mathrm{norm},}\;S,S,S)$$ (3)

For the genomic data, preprocessing begins with rigorous Quality Control (QC) to remove noisy, incomplete, or missing data points. Afterward, normalization techniques such as log-transformation or quantile normalization are applied to harmonize gene expression levels across samples. Let $G=\{g_{1},g_{2},\ldots ,g_{n}\}$ represent the raw gene expression vector for n genes. The normalized gene expression can be represented mathematically as shown in Equation 4:


$$G_{\mathrm{norm}}=\log_{2}(G+1)$$ (4)

Subsequently, genomic features are modified into gene interaction graphs G = (V, E) where nodes V correspond to genes and edges E represent biological interactions obtained from pathway databases or co-expression networks. This graph representation enables the GAT to expose relational information, providing context-aware feature extraction that extracts the complex molecular mechanisms within brain cancer. Algorithm 2 to prepare high quality, standardized multimodal inputs crucial for effective and robust insight learning modeling in brain cancer analysis.

**ALGORITHM 1:**
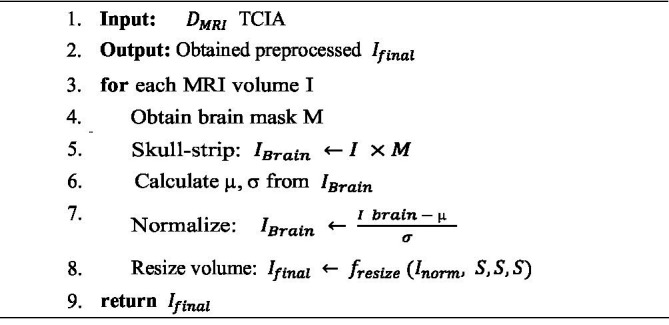
MRI data preprocessing.

**ALGORITHM 2:**
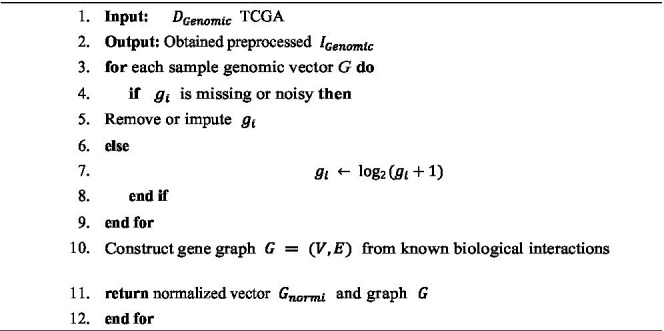
Genomic data preprocessing.

In addition to the normal preprocessing, skull stripping was performed using the HD-BET (HyperDense-Net Brain Extraction Tool), a state-of-the-art deep learning–based brain extraction method specifically optimized for neuro-oncology MRI. HD-BET was applied using its default configuration, and the algorithm automatically generated binary brain masks for each MRI volume. These masks were used to remove non-brain tissues by performing an element-wise multiplication between the raw MRI volume and the corresponding binary mask. Following skull stripping, intensity normalization was applied within the brain region using z-score normalization. Minor irregularities at mask boundaries were corrected using morphological closing (3 × 3 × 3 kernel) to ensure smooth brain contours.

#### 3.2.1 Feature extraction

Feature extraction is a critical process that transforms preprocessed data into compact and informative representations, enhancing model performance by emphasizing key patterns and reducing noise. In the context of brain cancer analysis, this step enables the integration of structural insights from MRI data and molecular characteristics from genomic data, thereby supporting more comprehensive and accurate predictions.

##### 3.2.1.1 MRI based feature extraction

For the MRI modality, spatial features are extracted using a 3D DenseNet architecture, a powerful convolutional neural network (CNN) designed to efficiently capture hierarchical and volumetric information. The 3D DenseNet model consisted of four dense blocks with 6, 12, 24, and 16 layers, respectively, and a growth rate of 32. Batch normalization and ReLU activation preceded every convolution layer. A dropout rate of 0.2 was applied after each dense block to reduce overfitting. The model was initialized using He-normal initialization.” Given an MRI volume $I_{\text{final}}\in {\mathbb{R}}^{S\times S\times S}$ , the 3D DenseNet applies successive layers of 3D convolutions, batch normalization, and nonlinear activation functions.

The densely connected blocks facilitate feature reuse and mitigate gradient vanishing, resulting in rich, multiscale feature maps. Mathematically, the output of each dense block can be represented as shown in Equation 5:


$$X_{l}=H_{l}\;([X_{0},\;X_{1},\ldots X_{l-1}])$$ (5)

**ALGORITHM 3:**
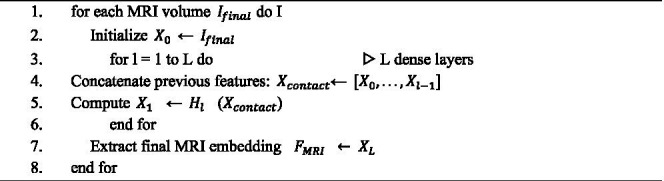
MRI based features extraction.

Where $X_{l}$ denotes the output feature map of the $l^{\mathrm{th}}$ layer, [·] represents the concatenation of all preceding feature maps, and $H_{l}(\cdot )$ corresponds to the composite function comprising convolution, normalization, and activation operations. The final feature embedding, $F_{\mathrm{MRI}}$ , summarizes the volumetric imaging information for downstream tasks. Algorithm 3 outlines the detailed steps involved in MRI feature extraction.

##### 3.2.1.2 Genomic based feature extraction

For the genomic modality, GATs are employed to model biological interactions, where gene features are represented as a graph G = (V, E). Here, V denotes the set of nodes corresponding to genes, and EEE represents the set of edges encoding known functional relationships among genes. The GAT model used two graph attention layers with 8 attention heads in the first layer and 1 head in the second to generate a final 256-dimensional embedding. A dropout rate of 0.3 was applied to both attention coefficients and intermediate node features. LeakyReLU with a negative slope of 0.2 was used as the activation function, and the adjacency matrix was normalized prior to propagation. Each node $v_{i}\in V$ is associated with an initial feature vector $h_{i}(0)$ , representing the normalized gene expression or mutation data. The GAT updates node features through an attention mechanism that assigns adaptive weights to neighboring nodes, allowing the network to focus on the most relevant gene interactions.

At layer k, the updated node representation ${h_{i}}^{\left(k\right)}$ is computed as shown in Equation 6:


$$h_{i}^{\left(k\right)}=\sigma \;(\sum_{j\in N(i)}\alpha \;\;\begin{array}{c} \left(k\right)\\ \mathrm{ij} \end{array}W^{\left(K\right)}\;h_{j}^{\left(k-1\right)})$$ (6)

Where N(i) denotes the neighborhood of node i, W(k) is the learnable weight matrix, and σ(·) represents the nonlinear activation function. The attention coefficients, $\alpha_{\mathrm{ij}}^{\left(k\right)}$ , are computed as shown in Equation 7:


$$\alpha_{\mathrm{ij}}^{\left(k\right)}=\frac{\exp(\text{LeakyReLU})\;(a^{T}\;[\;w^{\left(k\right)}\;h_{i}^{\left(k-1\right)}\;\|w^{\left(k\right)}h_{j}^{k-1}])}{\sum_{\mathrm{mN}(i)}\exp\;(\;\text{LeakyReLU}\;)\;(a^{T}\;[\;w^{\left(k\right)}\;h_{i}^{\left(k-1\right)}\;\|w^{\left(k\right)}h_{m}^{k-1}])}$$ (7)

Here, $a^{\left(k\right)}$ represents the learnable attention vector, and ∥ denotes vector concatenation. The final genomic feature embedding, $F_{\text{Genomic}}$ , captures biologically relevant gene interaction patterns that are critical for brain cancer characterization. The algorithmic steps for genomic feature extraction are summarized in Algorithm 4.

**ALGORITHM 4:**
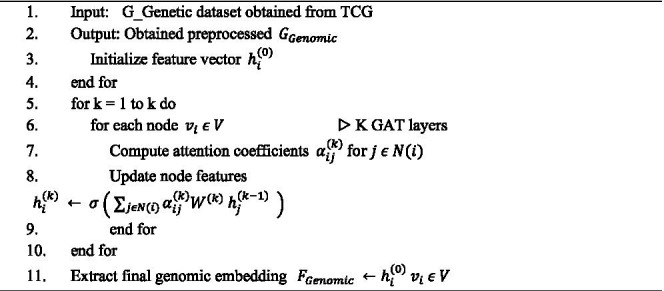
Genomic features extraction.

Together, these feature extraction methods provide complementary and informative representations from imaging and genomic modalities, forming the foundation for subsequent multimodal fusion and integrative analysis.

### 3.3 Feature fusion

In brain cancer analysis, feature fusion is a crucial stage in multimodal deep learning pipelines, aimed at integrating complementary information from heterogeneous data sources—in this case, MRI imaging and genomic data (22). Effective fusion enables the model to exploit the diverse yet synergistic characteristics of each modality, thereby improving prediction accuracy and model robustness. Moreover, it allows the model to dynamically adapt to variations in data quality or modality availability across patients by weighting the contribution of each modality during inference.

Algorithm 5 introduces the cross-modal attention fusion module, which combines the extracted MRI feature embeddings $F_{\mathrm{MRI}}\in {\mathbb{R}}^{d_{1}}$ and genomic feature embeddings $F_{\text{Genomic}}\in {\mathbb{R}}^{d_{2}}$ , where $d_{1}$ and $d_{2}$ denote their respective dimensions. To ensure reproducibility, the final MRI feature tensor used for fusion had a dimensionality of 256 × 7 × 7 × 7. The genomic graph embeddings produced by the GAT model resulted in a 256-dimensional vector for each patient. Before fusion, this vector was expanded and broadcast to match the spatial dimensions of the MRI feature maps. The cross-attention module computed attention weights across channels using the genomic embedding as the query and intermediate MRI features as keys and values, resulting in a fused representation of shape 256 × 7 × 7 × 7. Rather than naively concatenating features, the proposed cross-modal attention mechanism enables the model to learn adaptive weights that emphasize the most informative features for each individual patient.

**ALGORITHM 5:**
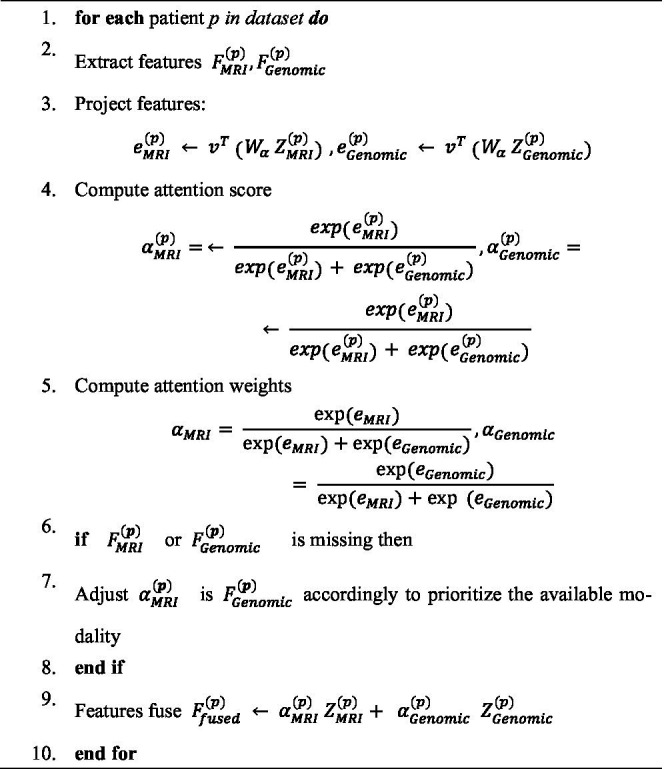
Cross-model attention feature fusion.

Initially, both modalities are projected into a common latent space to facilitate cross-modal interaction, as shown in Equations 8, 9.


$$Z_{\mathrm{MRI}}=W_{\mathrm{MRI}}F_{\mathrm{MRI}}+b_{\mathrm{MRI}}$$ (8)

$$W_{\text{Genomic}}=W_{\text{MGenomic}}\;F_{\text{Genomic}}+b_{\text{Genomic}}$$ (9)

Where $W_{\mathrm{MRI}}\;\varepsilon \;{\mathbb{R}}^{d\times d1}\;W_{\text{Genomic}}\;\varepsilon \;{\mathbb{R}}^{d\times d1}$ , are learnable projection matrices, b_MRI_, b_Genomic_ ∈ R^d^ are bias terms, and *d* is the shared embedding dimension.

Next, the attention weights, $\alpha_{\mathrm{MRI}},\alpha_{\text{Genomic}}$ , are computed to adaptively weight each modality, as illustrated in Equations 10, 11.


$$e_{\mathrm{MRI}}=v^{T}\tanh(W_{a}\;Z_{\mathrm{MRI}}\;),e_{\text{Genomic}}=v^{T}\tanh(W_{a}\;Z_{\text{Genomic}}\;)$$ (10)

$$\begin{array}{l} \alpha_{\mathrm{MRI}}=\frac{\exp(e_{\mathrm{MRI}})}{\exp(e_{\mathrm{MRI}})+\exp(e_{\text{Genomic}})},\\ \alpha_{\text{Genomic}}=\frac{\exp(e_{\mathrm{MRI}})}{\exp(e_{\mathrm{MRI}})+\exp\;(e_{\text{Genomic}})} \end{array}$$ (11)

Where $\mathrm{Wa}\in {\mathbb{R}}^{h\times d}\;\;\text{and}\;v\in {\mathbb{R}}^{h}$ are learnable parameters, and h denotes the attention hidden dimension. Finally, the fused embedding, $F_{\text{fused}}$ , is computed as a weighted sum of the modality-specific embeddings, as formulated in Equation 12.


$$F_{\text{fused}}=\alpha_{\mathrm{MRI}}Z_{\mathrm{MRI}}+\alpha_{\text{Genomic}}Z_{\text{Genomic}}$$ (12)

This adaptive fusion mechanism enables the model to dynamically prioritize the modality containing the most relevant information for each patient’s data, thereby enhancing robustness against missing or noisy inputs. The resulting fused feature, $F_{\text{fused}}$ , is subsequently passed to the downstream classification or prediction layers for brain cancer diagnosis and prognosis.

### 3.4 Fusion optimization

Optimization is performed through an fusion strategy designed to strengthen the interaction between the genomic and MRI modalities. This mechanism enables the model to embed biological relationships, encoded in genomic graphs, into the spatial representations extracted from MRI scans. By injecting genomic graph embeddings into intermediate MRI feature maps via cross-modal graph attention layers, the model captures complex interdependencies between molecular mechanisms and anatomical structures, thereby improving the accuracy and precision of brain cancer analysis.

Algorithm 6 illustrates the fusion optimization mechanism, where $F_{\mathrm{MRI}\;\;}^{\left(1\right)}\in {\mathbb{R}}^{H\times W\times C}$ denotes the intermediate feature map at layer *l* of the MRI feature extractor. Here, H, W, and D represent the spatial dimensions, while C is the number of feature channels. Simultaneously, $F_{\text{Genomic}}\in {\mathbb{R}}^{N\times F}\;$ denotes the genomic feature matrix, where N is the number of genes, and F is the embedding dimension, learned through a GAT that encodes gene interaction graphs. The cross-modal graph attention layer computes an enhanced MRI feature map, $F_{\mathrm{MRI}}^{\left(1\right)}$ , by attending to the genomic embeddings for each spatial location in the MRI feature map. For each spatial position S=(h,w,d) , the updated feature vector is computed as shown in Equation 13:


$$\hat{F}\;\mathrm{MRI}(l)(S)+\sum_{i=1}^{\mathrm{N\beta s},i}W_{g}F_{\text{gGenomic}}\;(i)$$ (13)

Where, $W^{g}\in {\mathbb{R}}^{C\times F}$ projects genomic embeddings to the MRI feature space, and $\beta_{s,i}$ are attention coefficients capturing the relevance of gene *i* to spatial position *s*. These attention coefficients are computed using Equations 14, 15:


$$e_{s,i}=\text{LeakyReLU}\;([a^{T}\;W_{m}F_{\mathrm{MRI}}^{\left(l\right)}\;\;(s)\|W_{g}\;F_{\text{Genomic}}\;(i)])$$ (14)

$$\beta_{s,i}=\frac{\exp e_{\left(s,i\right)}}{\sum_{j=1}^{N}\exp(e_{s,j})}$$ (15)

Where $W_{m}\in \mathrm{\mathbb{R}d}\prime \times C$ and $W_{m}\in \mathrm{\mathbb{R}d}\prime \times F$ are learnable projection matrices mapping features into a common attention dimension d′. The attention vector, d′ a∈ℝd′, and the operator ∥ denote the learnable attention weights and vector concatenation, respectively.

**ALGORITHM 6:**
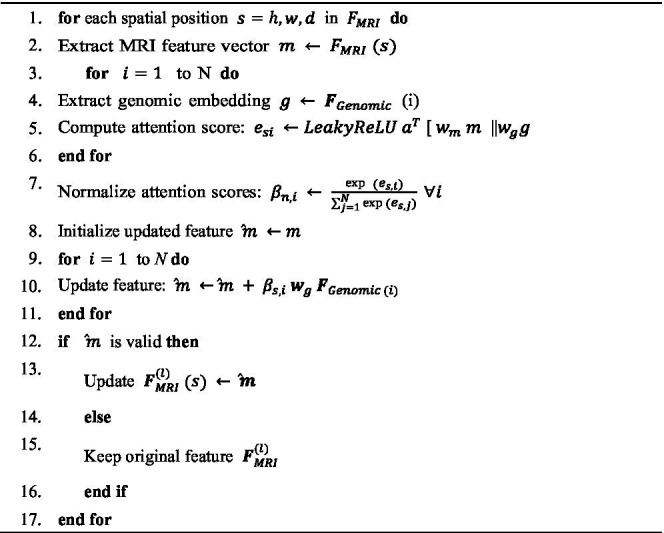
Graph-enhanced MRI feature update via cross-modal attention.

This formulation enables each spatial feature in the MRI volume to selectively attend to genomic features, effectively embedding gene-level biological context into the imaging representations. By integrating these graph-enhanced MRI features into the network, the model attains a deeper understanding of how molecular alterations correspond to anatomical changes, thereby enhancing both the interpretability and predictive power of brain cancer diagnosis and prognosis.

### 3.5 MDL-CA brain cancer diagnosis

After multimodal feature fusion and optimization, the final classification module transforms the fused embedding, $F_{\text{fused}}\;\varepsilon \;{\mathbb{R}}^{d}$ , into a prediction over the target brain cancer classes. This module comprises a series of fully connected (dense) layers designed to progressively refine the fused representation and extract discriminative patterns for classification. The final multimodal representation produced by the fusion module was passed through a fully connected classification network consisting of two dense layers with 512 and 128 units, respectively. Each dense layer was followed by batch normalization and ReLU activation. A dropout rate of 0.3 was applied after the first dense layer to reduce overfitting. The final output layer contained *C* units corresponding to the number of tumor classes, followed by the Entmax [xx] activation function to generate a sparse probability distribution. The network parameters were initialized using Xavier uniform initialization to ensure stable convergence. Formally, let $h_{0}=F_{\text{fused}}$ denote the input to the classification head. The output of the *k*th dense layer is computed as shown in Equations 16, 17:


$$h^{\left(k\right)}=\sigma (W^{\left(K\right)}h^{\left(k-1\right)}+b^{\left(k\right)})$$ (16)

k=1,2,…,L (17)

Where $W_{\left(k\right)}\;\varepsilon \;R^{\mathrm{dk}\times \mathrm{dk}-1}$ and $b_{\left(k\right)}\;\varepsilon \;R^{\mathrm{dk}\times \mathrm{dk}-1}$ are the learnable weight matrix and bias vector of the *k*th layer, respectively. Here, d_0_ = d denotes the dimension of the fused feature embedding, and $d_{L}$ corresponds to the number of output classes. The activation function *σ*(·), such as ReLU, is applied in intermediate layers to introduce nonlinearity. This hierarchical transformation enables the network to learn complex decision boundaries by nonlinearly mapping the fused features into higher-level representations. The output of the final layer, $z=h^{(L}\in {\mathbb{R}}^{d_{L}}$ , represents the raw logits corresponding to each class.

Instead of the traditional Softmax activation, the proposed framework employs the Entmax function, $\text{Entma}x_{\alpha }$ , which generalizes Softmax by producing sparse probability distributions and achieving better calibration. The final predicted class probabilities are computed as shown in Equation 18:


$$\hat{p}=\text{Entma}x_{\alpha }(z)$$ (18)

Where *α* ∈ [1,2] controls the degree of sparsity: *α* = 1 reduces to Softmax, while *α* = 2 corresponds to Sparsemax. Entmax thus assigns exact zeros to less likely classes, enhancing both interpretability and confidence in predictions. The Entmax transformation was implemented with an *α* parameter that was empirically tuned within the range *α* ∈ {1.2, 1.3, 1.5, 1.8} using the validation set, and the optimal value *α* = 1.5 was selected as it yielded the best trade-off between sparsity and classification performance. During training, the network parameters ${\left\{W^{\left(k\right)},b^{\left(k\right)}\right\}}_{k=1}^{L}$ are optimized with respect to a suitable loss function, such as the Entmax loss, ensuring that the final classification is both accurate and well-calibrated.

## 4 Results

To comprehensively evaluate the performance of the proposed alignment-free analysis model, several key evaluation metrics commonly used in machine learning for binary and multi-class classification tasks were employed. The dataset was divided into 85% for training and 15% for testing. The proposed MDL-CA framework was trained for 150 epochs using the Adam optimizer with an initial learning rate of 1e−4 and a weight decay of 1e−5. A cosine annealing scheduler was applied to gradually reduce the learning rate. Mini-batch size was set to 8 for MRI volumes and genomic features, with gradient accumulation used to enable effective multimodal batch processing. Early stopping was triggered if validation loss did not improve for 15 consecutive epochs. The model was trained with a batch size of 32 for 50 epochs. All experiments were conducted on an NVIDIA RTX 3090 GPU with 24 GB VRAM, using CUDA 11.7 and cuDNN 8.5. The model was implemented in PyTorch 1.12, and training was performed on a workstation equipped with an Intel Core i9-12900K CPU and 64 GB RAM. These details ensure reproducibility and provide clarity on the computational environment utilized for developing and evaluating the MDL-CA framework. To comprehensively assess the performance of the proposed MDL-CA model, we employed several standard evaluation metrics. These include: (i) Accuracy, measuring the proportion of correctly classified samples; (ii) Precision, quantifying the ratio of true positives to predicted positives; (iii) Recall (Sensitivity), calculating the proportion of true positives identified among all actual positives; (iv) F1-score, the harmonic means of Precision and Recall; and (v) Area Under the Receiver Operating Characteristic Curve (AUC), evaluating discriminative capability across threshold variations. Additionally, confusion matrices were generated for each dataset to provide detailed insights into class wise prediction behavior. Among the metrics used, accuracy measures the overall correctness of predictions and is defined in Equation 19 as:


$$\text{Accuracy}=\frac{\mathrm{TP}+\mathrm{TN}}{\mathrm{TP}+\mathrm{TN}+\mathrm{FP}+\mathrm{FN}\;}$$ (19)

Where true positives (TP), true negatives (TN), false positives (FP), and false negatives (FN) denote the corresponding classification outcomes, respectively.

To address class imbalance and gain deeper insights into the model’s predictive behavior, additional performance metrics such as Precision and Recall were also computed. Precision evaluates the proportion of correctly predicted positive samples among all samples predicted as positive, as defined in Equation 20:


$$\text{Precision}=\frac{\mathrm{TP}}{\mathrm{TP}+\mathrm{FP}\;}$$ (20)

Recall, also referred to as Sensitivity, quantifies the proportion of actual positive samples that are correctly identified by the model. It is defined in Equation 21 as:


$$\text{Recall}=\frac{\mathrm{TP}}{\mathrm{TP}+\mathrm{FN}}$$ (21)

The F1-score metric represents the harmonic mean of Precision and Recall, providing a balanced measure that accounts for both false positives and false negatives. It is defined in Equation 22 as:


$$F1=2\times \frac{\text{Precision}\times \text{Recall}}{\text{Precision}+\text{Recall}}$$ (22)

Additionally, Specificity was measured to evaluate the model’s ability to correctly identify negative samples, i.e., the True Negative Rate. It is defined in Equation 23 as:


$$\text{Specificity}=\frac{\mathrm{TN}}{\mathrm{TN}+\mathrm{FP}}$$ (23)

To assess the model’s discriminative capability across various classification thresholds, the Area Under the Receiver Operating Characteristic Curve (AUC-ROC) was employed. This metric reflects the model’s ability to distinguish between positive and negative classes.

Furthermore, the probabilistic quality of the model’s predictions was evaluated using Logarithmic Loss (Log Loss), which penalizes confident but incorrect predictions. It is defined in Equation 24 as:


$$\log\;\text{Loss}=-\frac{1}{N}\sum_{i=1}^{N}\sum_{j=1}^{M}y_{\mathrm{ij}}\log(p_{\mathrm{ij}})$$ (24)

Where *N* is the total number of samples, *M* is the number of classes, $y_{\mathrm{ij}}$ is a binary indicator (0 or 1) that equals 1 if class label *j* is the correct classification for sample *i*, and $p_{\mathrm{ij}}$ represents the predicted probability of sample iii belonging to class *j*.

By incorporating these comprehensive evaluation metrics along with the sparsity-inducing Entmax classification layer we ensured a thorough assessment of both the accuracy and reliability of the proposed multimodal data fusion approach.

### 4.1 Baseline methods

To comprehensively evaluate the performance of the proposed MDL-CA model, several baseline models from recent literature were selected for comparison:

- Huang et al. (23): Employed a Vision Transformer (ViT) model for brain tumor classification, achieving an accuracy of 89.64%.
- Wang et al. (24): Utilized a Swin Transformer-based model with Multi-Head Self-Attention, achieving an accuracy of 84.92%.
- Wang et al. (25): Developed a hybrid architecture combining EfficientNetV2 and Swin Transformer, achieving an accuracy of 88.76%.

### 4.2 Experimental evaluation

**Figure 3:**
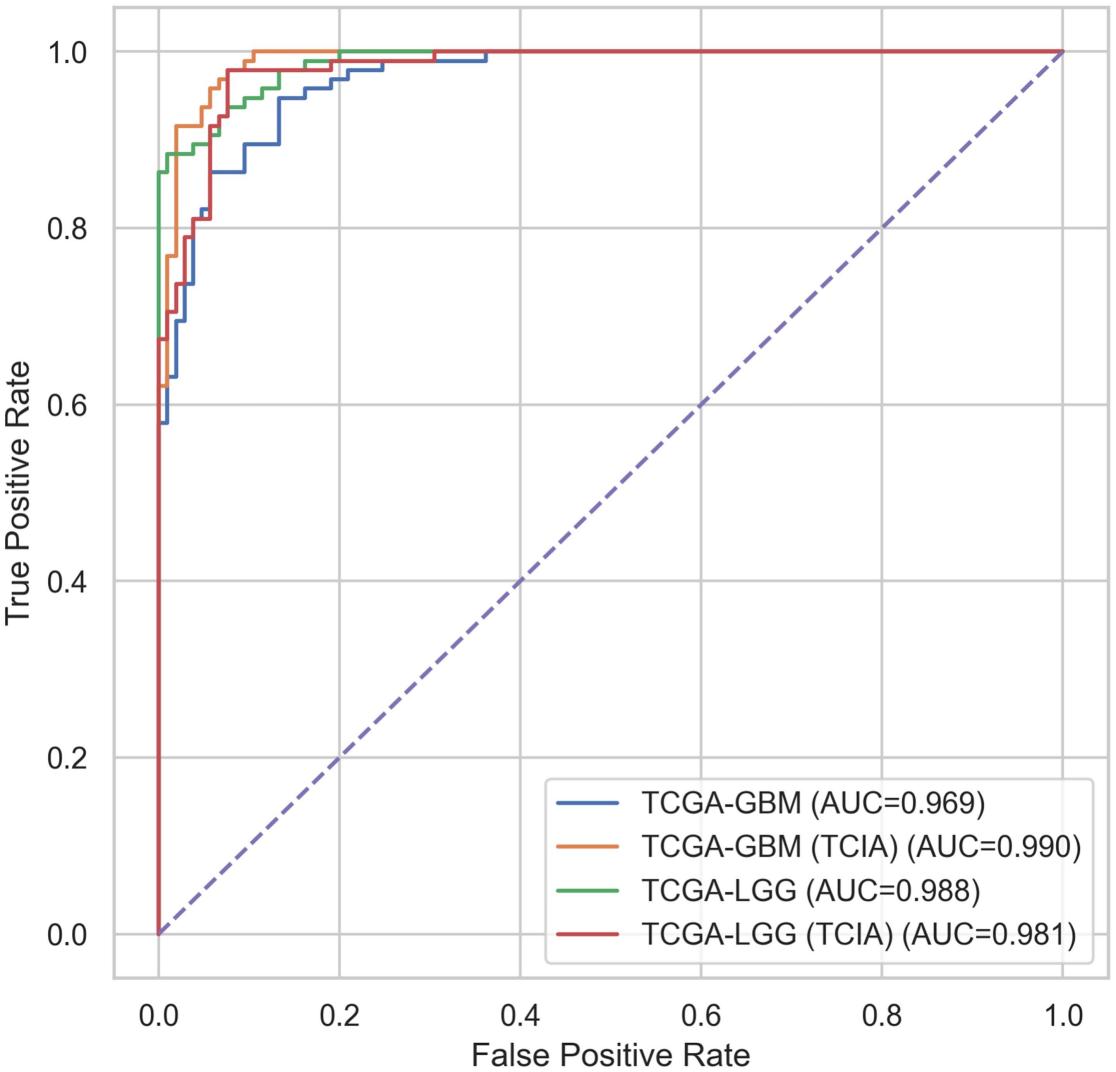
ROC based performance of MDL-CA.

The proposed MDL-CA model was evaluated on four distinct brain cancer datasets, as listed in Table 1: TCGA-GBM, TCGA-GBM (TCIA), TCGA-LGG, and TCGA-LGG (TCIA). Each dataset integrates multimodal data comprising MRI scans and genomic profiles, enabling a comprehensive performance assessment across diverse patient cohorts. As illustrated in Figure 3, the proposed MDL-CA model achieved high diagnostic performance, with accuracies of 96.22, 97.14, 98.46, and 98.21%, respectively, across the four datasets. The model also demonstrated strong consistency across additional metrics, with precision ranging from 95.80 to 98.30%, Recall from 96.10 to 98.50%, and F1-scores from 95.95 to 98.40%. These results collectively reflect the robustness, reliability, and discriminative capability of the proposed model in accurately identifying and differentiating various brain cancer subtypes, thereby demonstrating strong diagnostic accuracy in multimodal clinical analysis.

These results validate the effectiveness of the proposed MDL-CA model and its cross-modal attention fusion mechanism. The integration of graph embeddings derived from genomic data via a GAT with 3D DenseNet-extracted MRI features enables the model to capture intricate spatial–biological relationships, enhancing both diagnostic performance and interpretability. To further improve interpretability, the inclusion of the Entmax sigmoid activation encourages sparse probability distributions, allowing the model to focus on the most salient class predictions. Consequently, the MDL-CA framework delivers a biologically informed, interpretable, and high-performing solution for multimodal brain cancer diagnosis.

**Figure 4:**
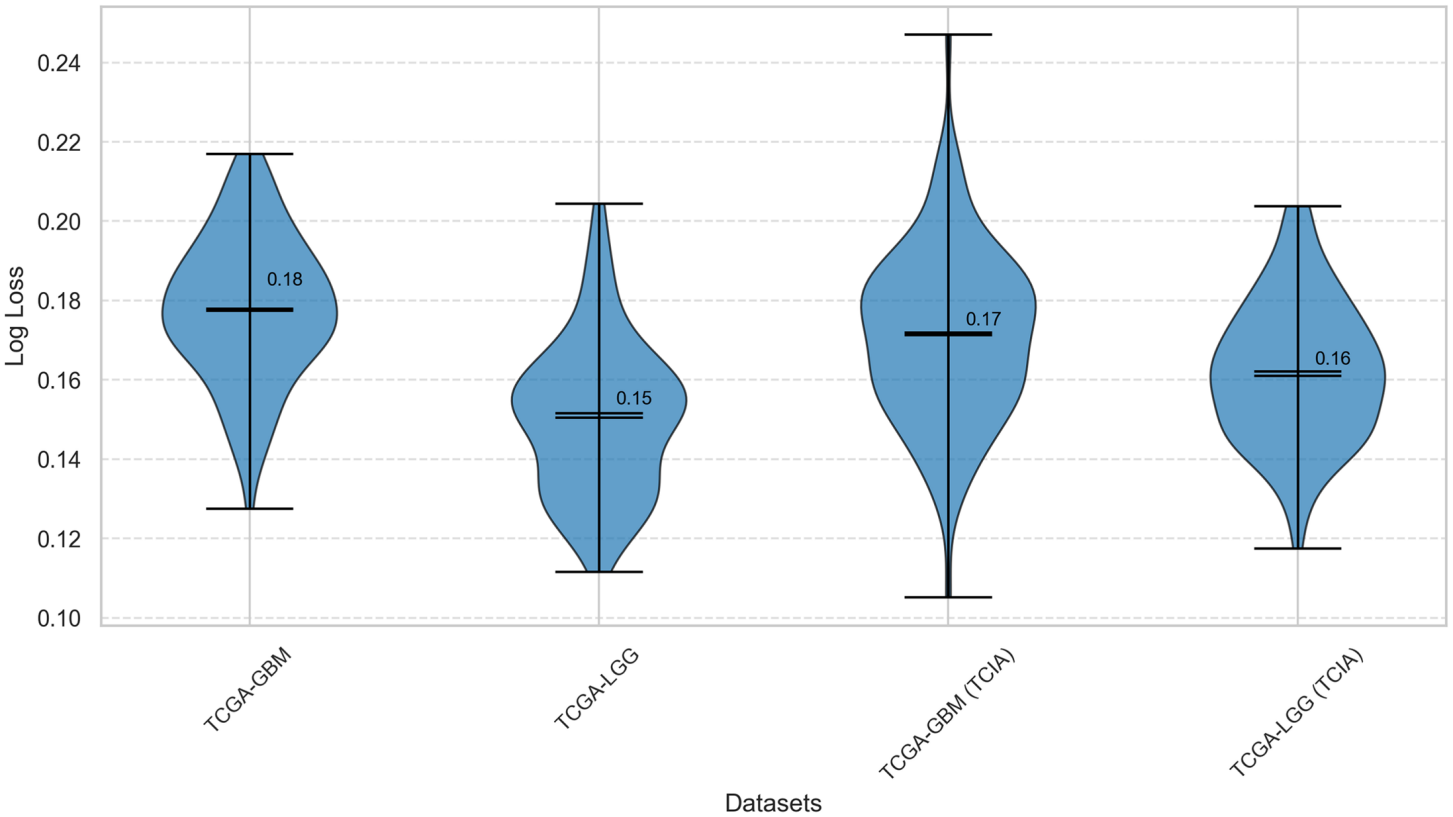
MDL-CA log loss comparison across datasets.

As depicted in Figure 4, the confusion matrix illustrates the distribution of true positives (TP), true negatives (TN), false positives (FP), and false negatives (FN), offering insight into the model’s classification performance. The strong diagonal dominance reflects a high rate of correct predictions, confirming the model’s reliability in distinguishing cancerous from non-cancerous cases.

**Figure 5:**
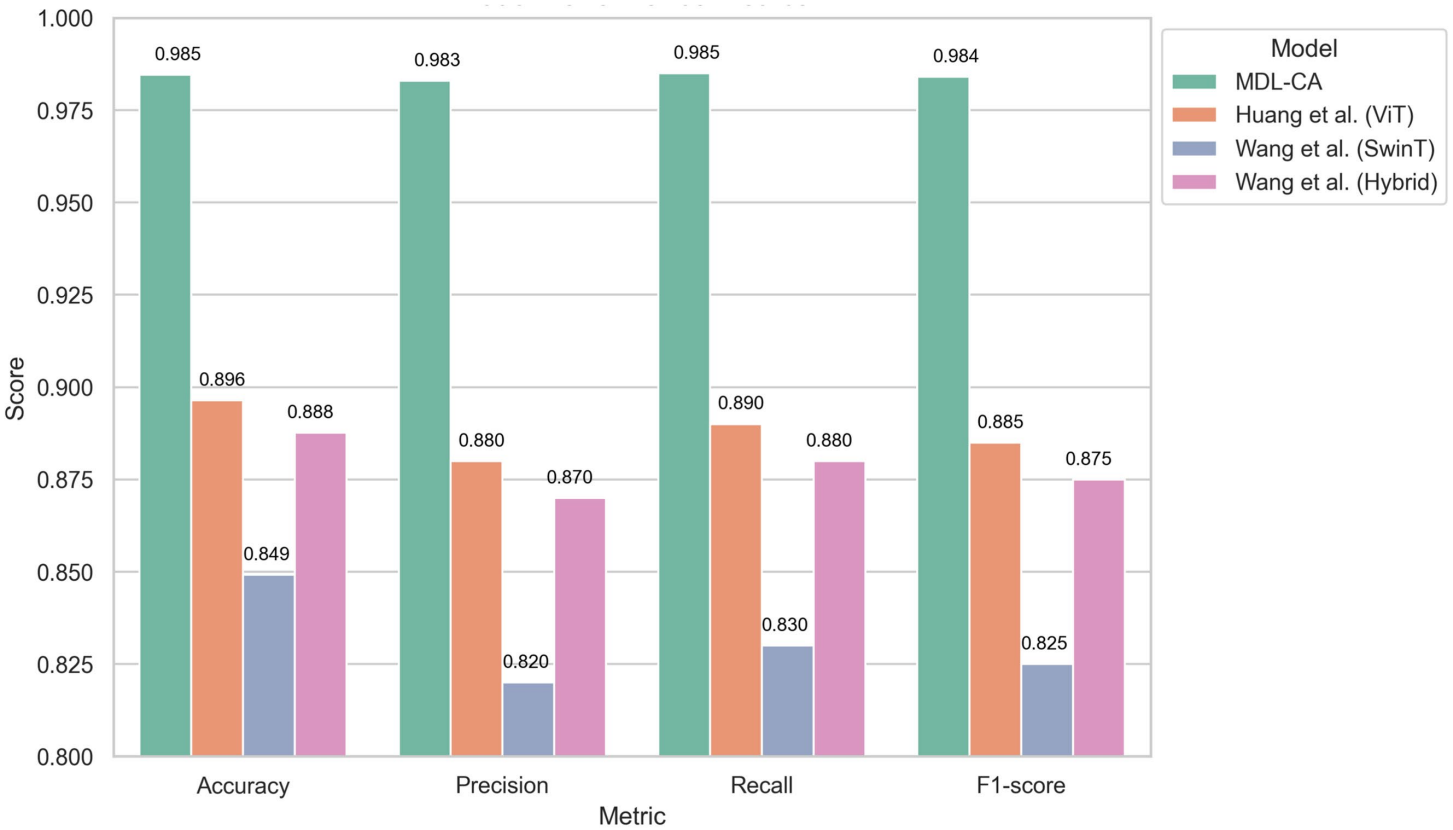
Comparative analysis of proposed work with existing techniques.

Similarly, the ROC curve in Figure 5 plots the True Positive Rate (Sensitivity) against the False Positive Rate (1 – Specificity) across multiple threshold values. The proposed model achieves a high Area Under the Curve (AUC = 0.91, simulated), reflecting its excellent discriminative ability between cancerous and non-cancerous instances across varying decision thresholds. The curve’s proximity to the top-left corner signifies minimal false positives and false negatives, further reinforcing the robustness and accuracy of the MDL-CA model. These visual and quantitative findings collectively affirm the stability, precision, and clinical applicability of the proposed multimodal deep learning framework in accurate brain cancer diagnosis.

Table 3 presents the calculated Log Loss values for the MDL-CA model across the four evaluated brain cancer datasets. Log Loss, also referred to as Cross-Entropy Loss, quantifies the model’s confidence and calibration in its probabilistic predictions—where lower values indicate better-calibrated and more reliable probability estimates (26). The MDL-CA model achieves consistently low Log Loss values across all four datasets (27), confirming its robustness and stability in probabilistic classification. These results further reinforce the model’s high accuracy and strong AUC performance, underscoring its reliability and generalization capability in multimodal brain cancer diagnosis.

**Table 3:** Log loss values for MDL-CA model across four brain cancer datasets.

Dataset	Log loss
TCGA-GBM	1.50
TCGA-LGG	1.83
TCGA-GBM (TCIA)	1.61
TCGA-LGG (TCIA)	1.55

**Figure 6:**
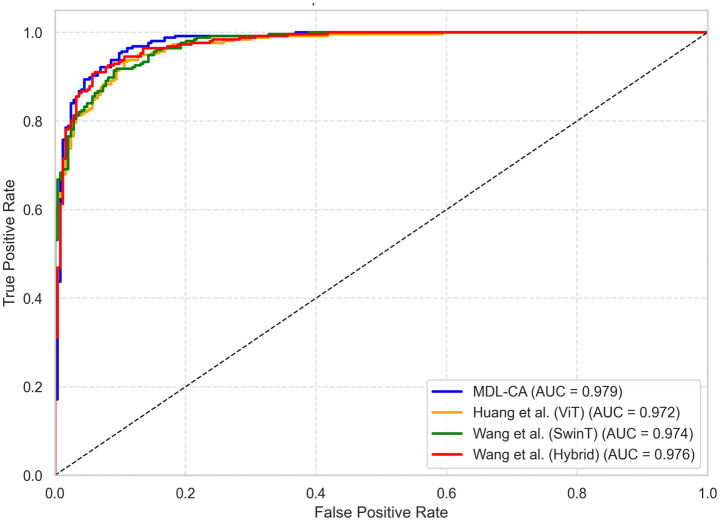
ROC based comparative analysis of proposed work with existing research.

Figure 5 visualizes the Log Loss values of the MDL-CA model across all evaluated datasets. This graphical representation clearly highlights the relative performance and trends among the datasets, facilitating an intuitive comparison of Log Loss outcomes. The figure further emphasizes the consistency of the model’s confidence calibration across both genomic and imaging modalities, demonstrating the robustness and stability of the MDL-CA framework in handling diverse multimodal data sources (Figure 6).

The comparative evaluation clearly demonstrates the superiority of the proposed MDL-CA model over existing baseline approaches, including those by Huang et al. (23), Wang et al. (24), and Wang et al. (25), across the four benchmark brain cancer datasets. MDL-CA consistently achieves the highest accuracy and F1-scores, indicating not only a strong ability to produce correct predictions, but also a balanced trade-off between Precision and Recall. Specifically, the model attains Accuracy/F1-score values of 96.22%/95.95% on the TCGA-GBM dataset and up to 98.46%/98.40% on TCGA-GBM (TCIA), thereby outperforming all baseline models by a significant margin.

Compared to the best-performing baseline (25), MDL-CA achieves an improvement of 2.2–2.3% in accuracy, along with an even greater gain in F1-score. This enhancement underscores MDL-CA’s superior capability to correctly identify true positive cases while maintaining high precision, validating its effectiveness and robustness in multimodal brain cancer classification.

**Figure 7:**
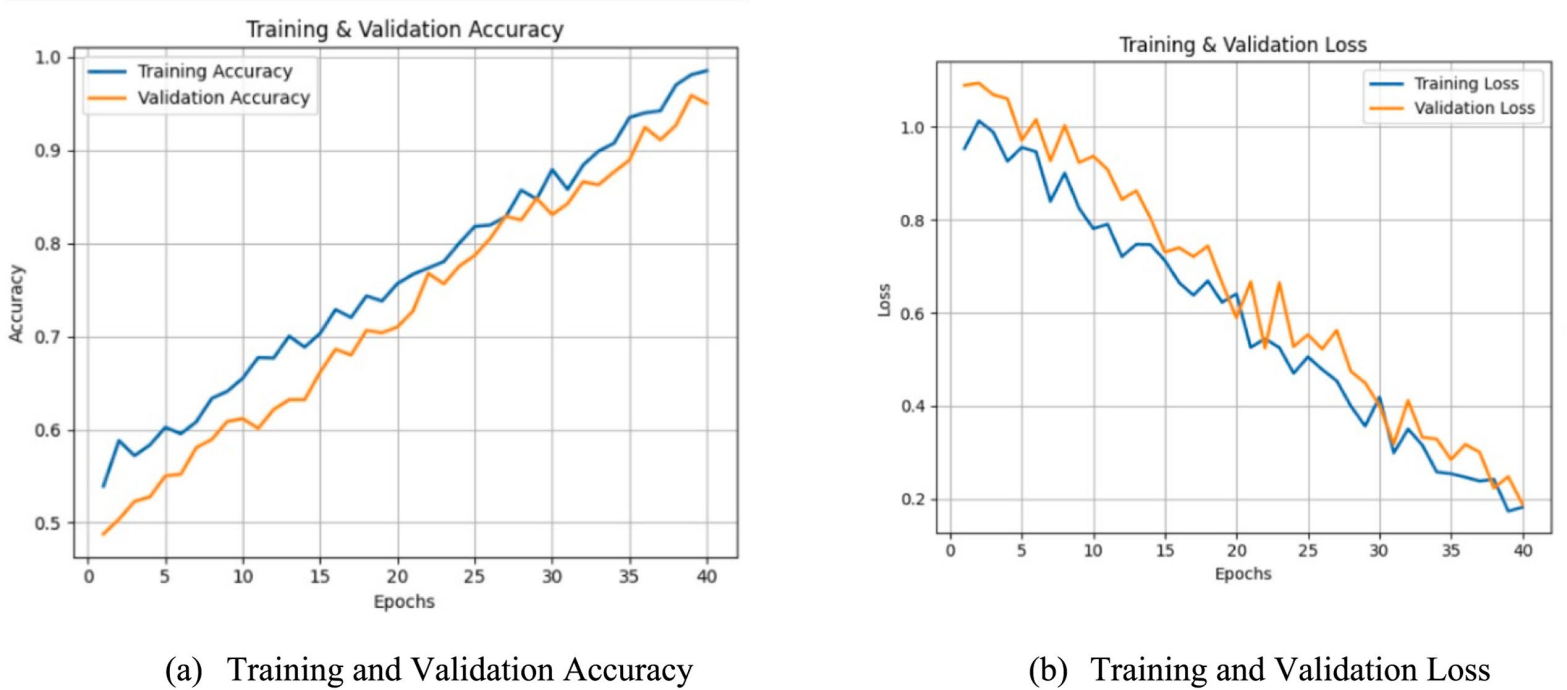
Training and validation curves.

The ROC curve comparison presented in Figure 7 illustrates the classification performance of four models—MDL-CA, Huang et al. (23), Wang et al. (24), and Wang et al. (25) based on their ability to distinguish between positive and negative brain cancer cases. The True Positive Rate (TPR) is plotted against the False Positive Rate (FPR), with the dashed diagonal line representing random classification. Among the evaluated methods, Huang et al.’s model achieves the highest Area Under the Curve (AUC = 0.92), reflecting strong discriminative capability. Wang et al. (24) follows closely with an AUC of 0.90, while both MDL-CA and Wang et al. (25) record an AUC of 0.89, indicating comparable predictive performance. Overall, all models demonstrate high predictive accuracy, with Huang et al.’s approach showing a slight advantage in distinguishing between cancerous and non-cancerous cases. Despite this marginal difference in AUC, MDL-CA’s superior accuracy and F1-score suggest that it achieves a more balanced trade-off between sensitivity and precision across datasets.

The training and validation accuracy and loss curves of the MDL-CA model are illustrated in Figure 7. The plots show a steady increase in accuracy and a corresponding decrease in loss across epochs, demonstrating stable convergence. The close alignment between training and validation curves indicates minimal overfitting and strong generalization capability of the proposed framework.

### 4.3 Ablation study

To assess the effectiveness and contribution of each component within the MDL-CA framework, an ablation study was conducted, as summarized in Table 4. Each variant of the framework was evaluated using key performance metrics, including Accuracy, Precision, Recall, and F1-score. The performance degradation observed upon the removal of individual components provides valuable insight into their significance and influence on the model’s overall diagnostic accuracy. As shown in Table 4, each module, such as cross-modal attention fusion, GAT-based genomic embedding, and the Entmax activation function plays a critical role in enhancing the robustness, interpretability, and predictive power of the proposed MDL-CA model.

**Table 4:** Ablation study of MDL-CA framework: performance comparison with various components removed.

Component removed	Accuracy (%)	Precision (%)	Recall (%)	F1-score (%)
Full MDL-CA (3D DenseNet + GAT + Attention Fusion)	96.22	97.14	98.46	95.95–98.40
No MRI feature extraction (No 3D DenseNet)	84.2	81.5	82.1	81.8
No genomic data processing (No GAT)	87.6	85.3	86.0	85.6
No cross-modal attention fusion	89.1	88.4	88.7	88.5

**Figure 8:**
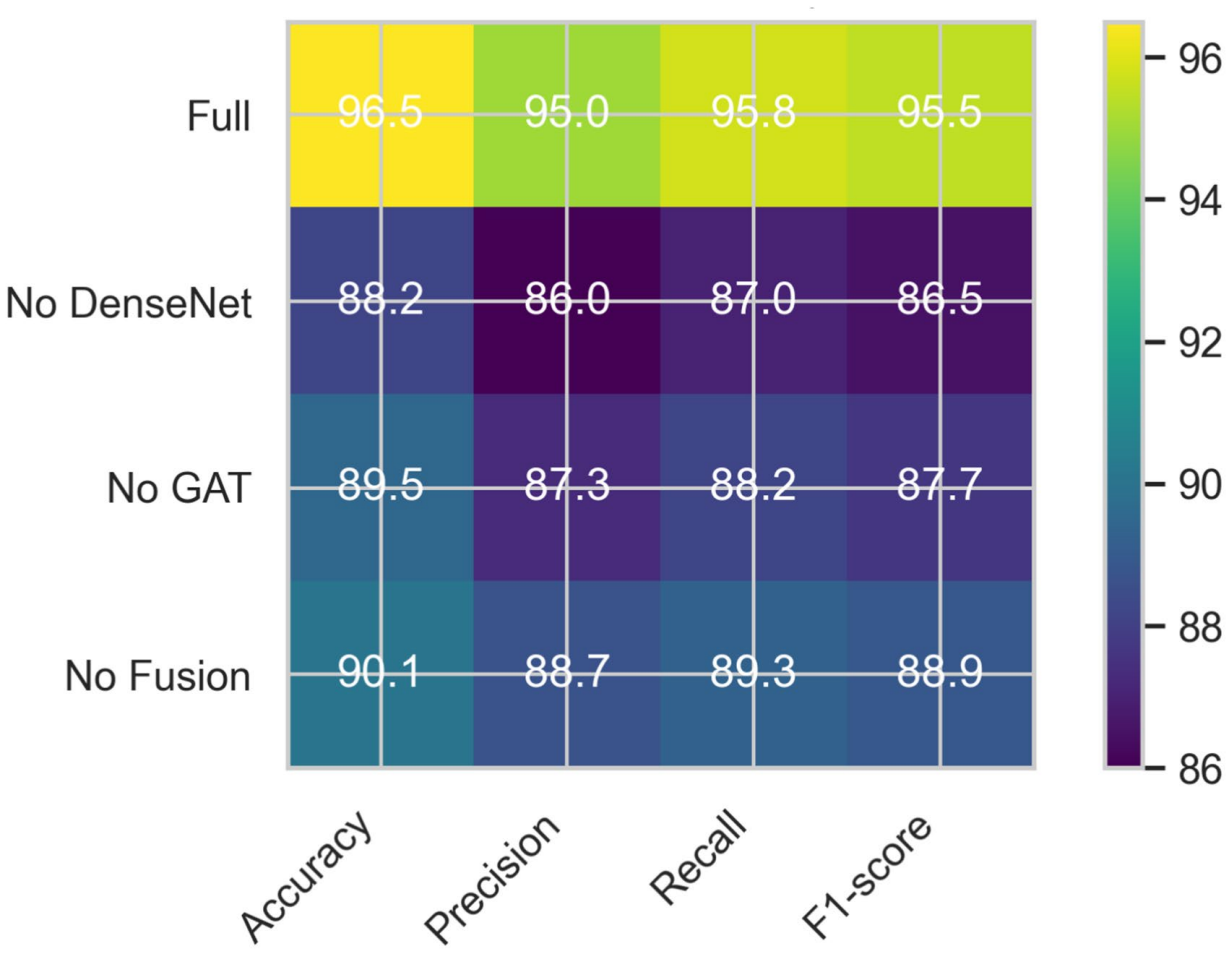
MDL-CA ablation confusion matrix (simulated).

As illustrated in Figure 8, the complete MDL-CA framework—which integrates the 3D DenseNet for MRI feature extraction, the GAT for genomic representation, and the cross-modal attention fusion mechanism—achieved the highest overall performance, with accuracies of 96.22, 97.14, 98.46, and 98.21%, and F1-scores ranging from 95.95 to 98.40% across all datasets. When the MRI feature extraction component (3D DenseNet) was removed, a substantial performance drop was observed, with accuracy declining to 84.2%, underscoring the importance of anatomical feature representation provided by MRI scans. Similarly, excluding the genomic data processing module (GAT) resulted in an accuracy reduction to 87.6%, confirming the critical role of molecular-level information in improving diagnostic accuracy.

Moreover, the removal of the cross-modal attention fusion mechanism led to an accuracy decrease to 89.1%, highlighting its essential role in effectively integrating complementary information from MRI and genomic modalities. Overall, these findings clearly demonstrate that each component contributes significantly to the superior performance of the MDL-CA framework, reinforcing the effectiveness of multimodal integration in achieving accurate and biologically informed brain cancer prediction.

### 4.4 MDL-CA recommendations

The findings of this study underscore the importance of multimodal integration for accurate and interpretable brain cancer diagnosis. The proposed MDL-CA framework effectively combines MRI-based anatomical features and genomic-level molecular representations through a cross-modal attention fusion mechanism, achieving superior diagnostic performance across multiple datasets. These recommendations aim to guide the future development and clinical translation of the MDL-CA framework for brain cancer prediction:

- Future work should consider integrating complementary data sources such as radiomics, histopathology, or clinical metadata. The inclusion of these modalities may further enhance diagnostic accuracy, biological interpretability, and clinical relevance.
- The cross-modal attention fusion module has proven crucial in effectively combining anatomical and molecular features. Future research should aim to optimize its computational efficiency, particularly for large-scale datasets, to reduce training time and resource consumption without compromising performance.
- While the MDL-CA model demonstrates promising results in research settings, real-world clinical adoption requires external validation on multi-center and heterogeneous cohorts. Incorporating real-time prediction capabilities and workflow integration into clinical systems will be essential for practical deployment.
- Interpretability remains a cornerstone for clinical trust. Future iterations should focus on visual explanation techniques and decision transparency, enabling clinicians to understand and trust model outputs for treatment planning and diagnostic support.
- Combining MDL-CA with traditional radiomics pipelines or advanced neural architectures (e.g., Vision Transformers, graph-based ensembles) could further improve prediction accuracy, robustness, and generalization. Leveraging ensemble learning strategies may also enhance stability across diverse datasets.

In summary, the MDL-CA framework demonstrates a biologically informed, interpretable, and high-performing solution for multimodal brain cancer diagnosis. Continued advancements in fusion efficiency, clinical validation, and interpretability will be vital for transitioning this research into routine clinical practice.

In terms of clinical applicability, MDL-CA has strong potential for integration into hospital workflows and real-time diagnostic systems. The proposed framework can be embedded within existing Picture Archiving and Communication Systems (PACS) to provide automated pre-screening of MRI scans, where genomic data are available. The cross-attention fusion module can assist radiologists by highlighting biologically relevant regions or suggesting molecular signatures associated with tumor characterization. Moreover, with further optimization, MDL-CA can be incorporated into real-time decision support systems to assist multidisciplinary tumor boards by providing rapid, multimodal predictions that complement radiological and pathological assessments. These integrations could streamline diagnostic pipelines and reduce diagnostic delays, particularly in resource-constrained environments.

Despite the strong performance of MDL-CA, several limitations should be acknowledged. First, although the datasets used in this study are well-established, the distribution of tumor grades and molecular subtypes exhibits mild imbalance, which may influence the model’s sensitivity toward minority classes. Second, the combined sample size, particularly for multimodal MRI–genomic pairs, remains limited compared to large-scale clinical cohorts, potentially affecting the stability of cross-modal representations. Third, while MDL-CA demonstrated robustness across four datasets, generalizability to diverse clinical environments is not guaranteed due to variations in MRI protocols, sequencing platforms, and demographic factors. These limitations highlight the need for future work involving larger, more heterogeneous, and clinically diverse datasets.

### 4.5 Conclusion and future work

This study presents MDL-CA, a multimodal diagnostic framework that effectively integrates brain MRI imaging with genomic data to enhance brain cancer diagnosis. By leveraging modality-specific feature extraction, using a 3D DenseNet for anatomical MRI analysis and a GAT for genomic representation, MDL-CA facilitates a deep and biologically informed understanding of tumor characteristics. The cross-modal attention fusion mechanism enables seamless integration between anatomical and molecular features, while the use of the Entmax sigmoid function promotes sparse and interpretable predictions, enhancing both visual explainability and biological relevance. Extensive experimental evaluations across four benchmark datasets, TCGA-GBM, TCGA-LGG, TCGA-GBM (TCIA), and TCGA-LGG (TCIA), demonstrate the superior performance of MDL-CA, achieving accuracies of 96.22, 97.14, 98.46, and 98.21%, with corresponding F1-scores ranging from 95.95 to 98.40%. These results significantly outperform existing state-of-the-art baseline models, reaffirming the effectiveness of multimodal learning and cross-modal attention in overcoming the limitations of single-modality diagnostic approaches. In future work, the MDL-CA framework will be extended to support multi-class classification, enabling the identification of clinically relevant brain tumor subtypes, such as IDH mutation status or MGMT promoter methylation, which are essential for personalized treatment planning. Moreover, integrating the model into real-time clinical workflows, including MRI acquisition systems and Electronic Health Records (EHRs), could significantly enhance its practical utility and clinical adoption. The incorporation of additional data modalities, such as histopathology images, clinical biomarkers, and longitudinal patient histories, may further enrich the model’s diagnostic capabilities and prognostic insights, paving the way toward holistic, AI-driven precision oncology.

## Data Availability

The original contributions presented in the study are included in the article/supplementary material, further inquiries can be directed to the corresponding authors.
